# Socioeconomic and education-based inequality in suspected developmental delays among Nepalese children: a subnational level assessment

**DOI:** 10.1038/s41598-023-31629-1

**Published:** 2023-03-23

**Authors:** Kiran Acharya, Md. Shafiur Rahman, Md. Rashedul Islam, Stuart Gilmour, Bibha Dhungel, Rajendra P. Parajuli, Tomoko Nishimura, Atsushi Senju, Kenji J. Tsuchiya

**Affiliations:** 1 New ERA, Rudramati Marg, Kalopul, Kathmandu, Nepal; 2 Research Centre for Child Mental Development, Hamamatsu University School of Medicine, 1-20-1 Handayama, Higashi-ku, Hamamatsu, 431-3192 Japan; 3 United Graduate School of Child Development, Osaka University, Kanazawa University, Hamamatsu University School of Medicine, Chiba University and University of Fukui, Osaka, Japan; 4 Department of Global Health Policy, The University of Tokyo, Tokyo, Japan; 5 National Cancer Center Institute for Cancer Control, Tokyo, Japan; 6 Graduate School of Public Health, St. Luke’s International University, Tokyo, Japan; 7 Department of Health Policy, National Centre for Child Health and Development, Tokyo, Japan; 8 Central Department of Zoology, Central Campus, Institute of Science and Technology (IOST), Tribhuvan University, Kritipur‑1, Kathmandu, Nepal

**Keywords:** Paediatrics, Public health, Epidemiology

## Abstract

Failure to meet early childhood developmental milestones leads to difficulty in schooling and social functioning. Evidence on the inequality in the burden of developmental delays across population groups, and identification of potential risk factors for suspected developmental delays (SDD) among younger children, are essential for designing appropriate policies and programs. This study explored the level of socioeconomic and maternal education-based inequality in the prevalence of SDD among Nepalese children at subnational level and identified potential risk factors. Individual-level data from the 2019 Nepal Multiple Indicator Cluster Survey was used to estimate the prevalence of SDD among children aged 3–4 years. Regression-based slope index of inequality (SII) and relative index of inequality were used to measure the magnitude of inequality, in terms of household socioeconomic status (SES) and mother’s education, in the prevalence of SDD. In addition, a multilevel logistic regression model was used to identify potential risk factors for SDD. The national prevalence of SDD was found to be 34.8%, with relatively higher prevalence among children from rural areas (40.0%) and those from Karnali Province (45.0%) followed by Madhesh province (44.2%), and Sudhurpashchim Province (40.1%). The prevalence of SDD was 32 percentage points higher (SII: −0.32) among children from the poorest households compared to their rich counterparts at the national level. At the subnational level, such inequality was found to be highest in Lumbini Province (SII = −0.47) followed by Karnali Province (SII = −0.37), and Bagmati Province (SII = −0.37). The prevalence of SDD was 36 percentage points higher (SII: −0.36) among children whose mother had no formal education compared to children of higher educated mothers. The magnitude of education-based absolute inequality in SDD was highest in Lumbini Province (SII = −0.44). Multilevel logistic regression model identified lower levels of mother’s education, disadvantaged SES and childhood stunting as significant risk factors for SDD. One in each three children in Nepal may experience SDD, with relatively higher prevalence among children from rural areas. Subnational level variation in prevalence, and socioeconomic and education-based inequality in SDD highlight the urgent need for province-specific tailored interventions to promote early childhood development in Nepal.

## Introduction

Early childhood development is an interactive process which involves the ordered and timely development of language, cognitive, motor, socio-emotional, and regulatory skills and capacities across the initial few years of life^1,2^. The vital domains of a child’s overall development include physical growth, socio-emotional development, readiness to learn, and literacy and numeracy skills, which build the basis for later life, and in particular set the path for health, learning, and well-being. Failure to meet early childhood developmental milestones in any of these domains leads to difficulty in schooling and social functioning, and has long-term health consequences^3^.

Early identification of suspected delays in achieving developmental milestones and timely implementation of effective interventions have been shown to minimize the long-term health and economic consequences, as well as improve later quality of life^4,5^. However, early identification of suspected developmental delays (SDD) in low- and middle-income countries (LMICs) remains a challenge, particularly because such screening is not a part of the routine health check-ups in the majority of these countries^6^. In LMICs, major progress has been made over the past decades to measure and reduce the prevalence of physical growth deficits and poverty. However, due to the weak and fragile health system, and cultural challenges of implementation of developmental surveillance in low-resourced settings, population-level data on cognitive and socioemotional development during early childhood have, until recently, remained limited^7^.

The United Nations Children's Fund (UNICEF) has developed an index comprising 10 items, known as the Early Childhood Development Index (ECDI), to assess the developmental status for children aged 3–4 years only^1^. Using this index, previous study reported that approximately one-third of children aged 3–4 years fail to meet basic cognitive and/or socioemotional milestones in LMICs^7^, with higher prevalence among children from Sub-Saharan Africa and South Asia^8^. Among South Asian countries, Nepal exhibited relatively higher prevalence of SDD (35.1%) in a previous study^8^. Multicounty studies documented that prevalence of SDD varies within each country; the prevalence was found to be higher among poor children relative to their wealthier counterparts^8,9^. Such variations are relatively large among LMICs with higher prevalence of SDD^8,10,11^. Along with socioeconomic status (SES), which is a well-established risk factor for developmental delays^12,13^, other factors such as gender, place of residence, and maternal education could affect the prevalence of SDD^9,14,15^.

As part of the recently adopted Sustainable Development Goals (SDGs), member states of the United Nations including Nepal have committed, as SDG target 4.2, to ensure quality early childhood development for all girls and boys by 2030^16^. Thus, identification of population groups at risk for SDD among Nepalese children, where the burden is relatively higher, could support Nepalese progress towards the SDGs^17^. Moreover, identification of potential determinants of SDD might reduce the knowledge gap. Previous studies reported consistent association of stimulating home environment, assessed by Home Observation for Measurement of Environment (HOME) scale score, with mental development index (MDI) at 24 and 36 months in Terai Nepal^18,19^. In addition, a recent study reported delayed gross motor development among girls aged 3 years who underwent delayed cord clamping^20^. Yet, to our knowledge, studies identifying potential risk factors of SDD among younger Nepalese children using wide range of covariates are limited^18–21^.

Most of the previous studies evaluated the level of inequality in prevalence of SDD at the national level and reported large socioeconomic disparity in prevalence of SDD^7–9^. However, those studies ignored subnational level assessments of such inequality. Recent studies reported marked subnational level differences in early childhood education and home stimulation^22^ as well as in access to health services^23^ and child health^24,25^ and nutrition indicators^26^ in Nepal. Moreover, the level of poverty and education is quite different across provinces in Nepal^27^. Taken together, we hypothesize that the level of socioeconomic and education-based inequality in the prevalence of SDD among children will vary across subnational regions.

Therefore, we aimed to explore the level and magnitude of inequality in the prevalence of SDD, in terms of household SES and maternal education, among Nepalese children at national and subnational levels. We additionally identified potential risk factors associated with SDD, and explored the subnational variation in the prevalence of SDD after adjusting for identified risk factors.

## Methods

### Data sources

This study analyzed data from the 2019 Nepal Multiple Indicator Cluster Survey (NMICS), which is a nationally representative population-based cross-sectional survey^22^. The survey was conducted by the Central Bureau of Statistics (CBS) with technical and financial support from the UNICEF. The main aim of the survey was to monitor the situation of women and children by capturing information on health, education, social protection, environment, and domestic violence along with an exhaustive set of socioeconomic, demographic, and geographic characteristics at the individual and household level.

### Study participants

The 2019 NMICS used a multistage stratified probability sampling technique, where stratification was used to establish a representative sample of households at the national and province level by separating each province into urban and rural areas. The urban and rural areas within each province were identified as the main sampling strata. The detailed methodology, sampling techniques, and questionnaires can be found elsewhere^22^. In summary, a total of 12,800 households from 512 enumeration areas (EAs) were selected for the survey, of which 12,655 households were successfully interviewed with a response rate of 99.7%. The 2019 NMICS initially identified 6749 children under 5 years of age, of which 6658 participated in the study. After excluding 3788 children aged 0–2 years (due to the scope of the current study), 2898 children aged 3–4 years (36–59 months) (weighted number: 2870 children) were selected for this study (see Additional File, Fig. S1).

### Outcome measures

The primary outcome of this study was SDD during early childhood, which was assessed using the ECDI. The ECDI is widely used in LMICs to assess whether a child is developmentally on track^2,7–9,28^, and estimated based on selected milestones that children are expected to achieve by ages 36 and 59 months^1^. The ECDI is constructed based on a mother- or caregiver-reported 10-item instrument (2 items for learning/cognition, 3 for literacy-numeracy, 2 for physical, and 3 for social-emotional domain)^1^. Details of the items are presented in the Supplementary Appendix (see Additional File, Method S1). A child is considered as developmentally on track if he/she is on track in at least three of the four major domains: learning/cognition, literacy-numeracy, physical, and social-emotional domain. In contrast, if a child is not on track for at least two domains, he/she will be considered as developmentally not on track or having SDD^1^.

### Covariates

Based on the conceptual models provided in the existing literature^15,29^, we have divided the covariates into four broad characteristics; child characteristics, parental characteristics, household characteristics, and community characteristics. Child characteristics included age (36–47 months, 48–59 months), sex (boys, girls), birth order (1, 2–4, ≥ 5), and being stunted (defined as a child having height-for-age Z-score lower than two standard deviation (−2SD) from the median of the WHO reference population^30^; no, yes). Parental characteristics included age (15–24 years, 25–34 years, ≥ 35 years), educational status and functional difficulties of mother (no, yes). Mother’s education was categorized as no education, basic or primary education (Grade 1–8), secondary education (Grade 9–12) and higher education (bachelor, masters or above). Household characteristics included household SES, household size (1–4 persons, 5–6 persons, > 6 persons), and iodine level of salt consumed in the household (None [0 ppm], inadequate [< 15 ppm], adequate [≥ 15 ppm]). Details of the household SES estimation are presented in the Supplementary Appendix (see Additional File, Method S2). We considered iodine level of salt consumed in the household as a proxy of inadequate iodine intake, which is considered to be a risk factor for adverse child development^31^. Community-level characteristics included place of residence (urban, rural), and province (Province 1, Madhesh province, Bagmati Province, Gandaki Province, Lumbini Province, Karnali Province, Sudurpashchim Province).

### Statistical analysis

Frequency distribution and descriptive analysis were used to describe participant characteristics. The prevalence of SDD, along with 95% confidence intervals (CI), was reported after adjusting for sampling weights. We assessed socioeconomic and education-based inequality in the prevalence of SDD at the national and subnational levels. We double-stratified household SES by dividing households into quintiles based on wealth scores separately for urban and rural areas. The magnitude of absolute inequality was measured using the slope index of inequality (SII) and the magnitude of relative inequality was assessed using the relative index of inequality (RII) and concentration index^32–37^. The SII is a regression-based measure of absolute inequality that assesses the difference—in terms of percentage points—in the estimated prevalence of SDD between the lowest/most-disadvantaged and highest/most-advantaged subgroups (for example, between the lowest wealth quintile and highest wealth quintile; between non-educated and higher educated), while taking into account of all subgroups of the ordered inequality dimension such as household SES and education^32,33,36^. On the other hand, the RII is a regression-based measure of relative inequality that presents the ratio of the estimated prevalence of SDD between the lowest/most-disadvantaged and highest/most-advantaged subgroups, while taking into account of all subgroups^32,33,36^. In order to estimate the SII and RII, first the weighted sample of the whole population is ranked based on their position in the cumulative distribution of inequality dimension (such as household SES, education), where the rank 0 is assigned to the most-disadvantaged or lowest subgroup and the rank 1 is assigned to the most-advantaged or highest subgroup. Then, the mid-point of range of the cumulative population distribution for each subgroup is estimated. Later, the outcome variable is regressed against the midpoint value for subgroups using logistic regression and the predicted values for the outcome of interest are then estimated for the most-disadvantaged subgroup (${v}_{0}$) and most-advantaged subgroup (${v}_{1}$). The SII is then estimated as, $SII={v}_{1}-{v}_{0}$ and the RII is estimated as, $RII={v}_{1}/{v}_{0}$. The SII and RII values present the magnitude of inequality. Any positive value of SII indicates that the indicator is concentrated among advantaged subgroup (pro-rich inequality in case of household SES), while negative value of SII indicates that the indicator is concentrated more among disadvantaged subgroup (pro-poor inequality). The RII takes only positive values; RII > 1 indicates that the indicator is more concentrated among the most-advantaged group than the most-disadvantaged group, while the RII values between 0 and 1 indicates the indicator is more concentrated the most-disadvantaged group. The relative concentration index (RCI) is a disproportionality measure (but not a regression-based measure) of relative inequality, which indicates to what extent the outcome of interest in concentrated among the most-advantaged or dis-advantaged groups^38^. The RCI is related to the Gini coefficient and can be presented in the form of a concentration curve^34^. The concentration curve plots the cumulative percentage of individual ranked based on their position in the distribution of inequality dimension on the x-axis and the cumulative percentage of the outcome variable on the y-axis. A diagonal line in such curve indicates the line of equality. The RCI is estimated as twice the area between the diagonal line (line of equality) and the concentration curve for that outcome^37,38^. The RCI values range from −1 to + 1. Similar to the SII value, a negative RCI value indicates that the outcome is concentrated towards the most-deprived subgroups and vice-versa. Details of these methods are presented elsewhere^32,33,36^. We used a multivariable logistic regression model to identify the determinants of SDD. Since participants of the NMICS 2019 were nested within households, and households were further nested within communities, we used a multilevel logistic regression analysis with a random intercept term at household and community level. We compared the random-effects model with a fixed-effect model using the likelihood ratio test^39^. Both unadjusted and adjusted models were used to provide odds ratios (OR) along with 95% CI. In addition, we estimated adjusted prevalence of SDD, adjusted for identified risk factors, to explore how risk factors contributed to the variation in the prevalence of SDD across provinces. Data management and statistical analysis were performed using Stata (MP version 16.1; StataCorp) and figures were prepared in R (Version 4.1.0).

### Ethics statements

Since this study used publicly available secondary datasets, ethical approval was not required from conducting this study. However, the survey implementation authority obtained necessary ethical approval from the respective organization. Verbal consent was obtained by the survey authority from each respondent prior to the interview.

## Results

### Participant characteristics

Table 1 presents the background characteristics of the study participants. A total of 2,870 children aged 3–4 years were included in this study, of whom around half (51.8%) were boys. About 36% of the children were stunted. Around half of the included children’s mothers were not educated (49.1%), whereas only 6.6% were higher educated. Only one percent of mothers had some kind of functional difficulty. About 76% of households consumed adequately iodized salt (≥ 15 ppm). Approximately two-thirds of children (65.3%) were from urban areas.

**Table 1:** Prevalence of suspected developmental delays among Nepalese children (N = 2870).

Characteristics	N (%)	Prevalence of suspected developmental delays (95% CI)	p-value^a^
Total	2870	34.8 (32.3–37.4)	
Child characteristics			
Age of children			
36–47 months	1468 (51.1)	42.2 (38.8–45.7)	** < 0.001**
48–59 months	1402 (48.9)	27.0 (23.9–30.4)	
Sex of children			
Boys	1487 (51.8)	35.2 (32.0–38.6)	0.703
Girls	1384 (48.2)	34.4 (31.0–37.9)	
Birth order			
1	713 (25.5)	24.7 (20.4–29.6)	** < 0.001**
2–4	1871 (66.8)	38.2 (35.0–41.4)	
≥ 5	215 (7.7)	42.4 (34.0–51.3)	
Stunting			
No	1818 (64.3)	30.2 (27.3–33.3)	** < 0.001**
Yes	1008 (35.7)	42.3 (38.4–46.4)	
Early childhood education			
Attending	1782 (97.3)	25.2 (22.7–27.8)	** < 0.001**
Not attending	50 (2.7)	54.9 (37.8–70.9)	
Parent’s characteristics			
Mother’s age			
15–24 years	746 (26.5)	32.9 (28.8–37.3)	0.494
25–34 years	1641 (58.4)	35.2 (31.9–38.7)	
35 years and above	425 (15.1)	37.4 (31.1–44.2)	
Mother’s education			
No education	816 (28.4)	49.1 (44.4–53.8)	** < 0.001**
Basic education	959 (33.4)	35.9 (31.9–40.2)	
Secondary education	905 (31.5)	25.0 (21.8–28.5)	
Higher education	190 (6.6)	14.5 (9.2–22.0)	
Functional difficulties of mother			
Yes	29 (1.0)	24.6 (13.9–39.8)	0.168
No	2781 (99.0)	35.1 (32.5–37.7)	
Household-level characteristics			
Household socio-economic status			
Poorest	652 (22.7)	46.9 (41.7–52.1)	** < 0.001**
Poorer	583 (20.3)	40.0 (34.0–46.3)	
Middle class	590 (20.6)	33.0 (28.7–37.7)	
Richer	573 (20.0)	31.8 (26.0–38.3)	
Richest	472 (16.4)	17.5 (12.9–23.4)	
Household size			
1–4 persons	1487 (54.2)	34.9 (31.8–38.2)	0.520
5–6 persons	817 (29.8)	33.2 (29.1–37.6)	
> 6 persons	441 (16.1)	37.3 (31.4–43.6)	
Iodine level in the salt consumed			
None (0 ppm)	151 (5.5)	32.4 (23.7–42.4)	0.804
Inadequate (< 15 ppm)	506 (18.5)	36.1 (30.0–42.7)	
Adequate (≥ 15 ppm)	2073 (75.9)	34.6 (31.7–37.6)	
Community-level characteristics			
Place of residence			
Urban area	1875 (65.3)	32.1 (28.8–35.5)	**0.002**
Rural area	995 (34.7)	40.0 (36.3–43.7)	
Province			
Province 1	465 (16.2)	23.1 (17.7–29.4)	** < 0.001**
Madhesh Province	707 (24.6)	44.2 (38.2–50.3)	
Bagmati Province	513 (17.9)	26.1 (21.6–31.2)	
Gandaki Province	195 (6.8)	20.5 (15.6–26.5)	
Lumbini Province	515 (18.0)	40.0 (33.5–46.8)	
Karnali Province	189 (6.6)	45.0 (37.4–52.8)	
Sudhurpashchim Province	285 (9.9)	40.1 (34.3–46.1)	

*CI* confidence interval, *ppm* parts per million.

^a^p-values were obtained through Chi-square test.

Significant values are in bold.

### Prevalence of SDD

**Figure 1:**
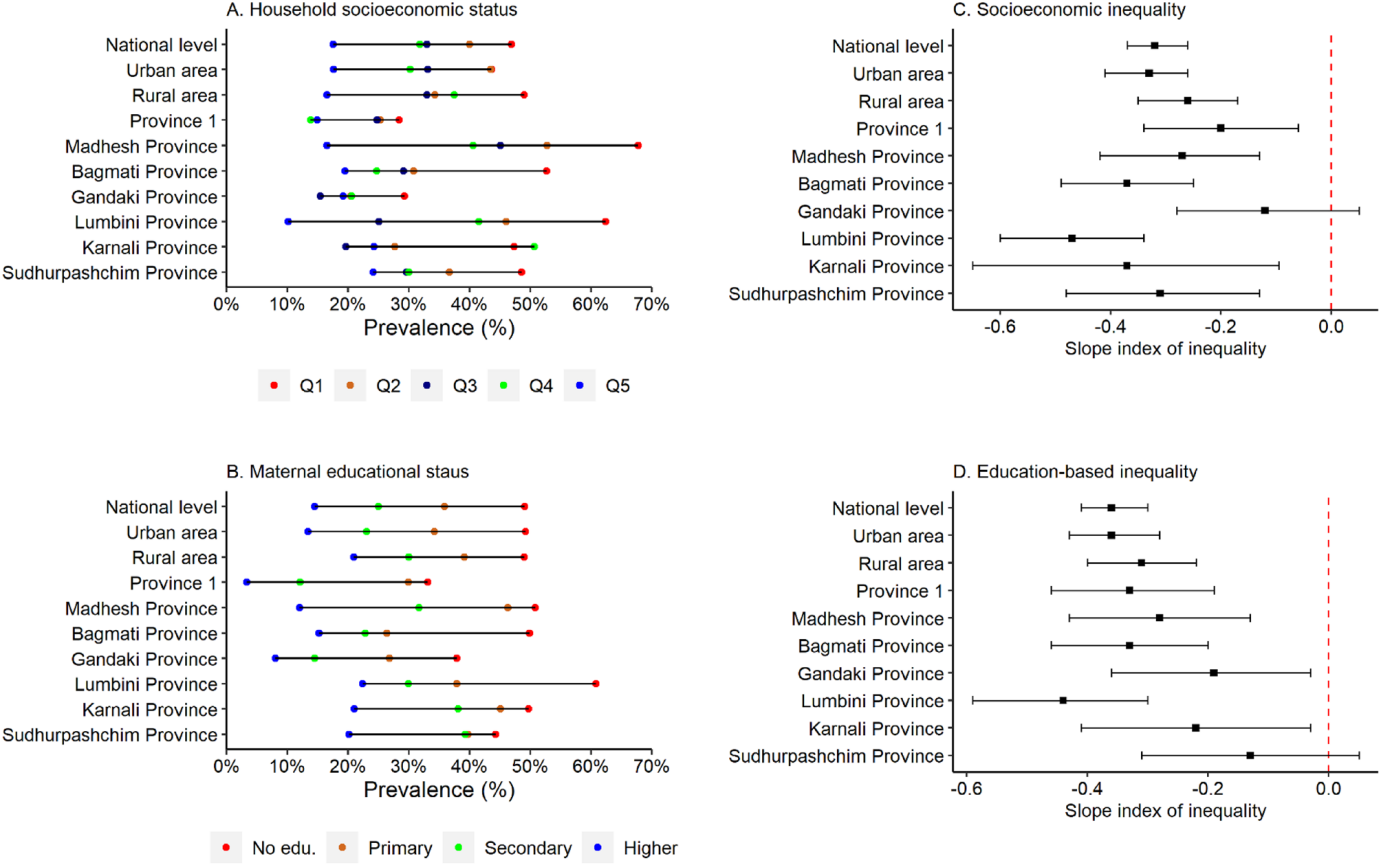
Prevalence of suspected developmental delays and level of absolute inequality among Nepalese children aged 3–4 years by household socioeconomic status and maternal education.

Overall, the prevalence of SDD among Nepalese children aged 3–4 years was 34.8% (Table 1), with relatively higher prevalence among children from rural areas (40.0%) than urban areas (32.1%) (Fig. 1). When estimated by domain, the prevalence of suspected delay was highest in the literacy-numeracy domain (59.6%), followed by the social-emotional (44.0%) and learning/cognition domains (9.7%) (see Additional File, Table S1). The prevalence of SDD was significantly higher among children aged 36–47 months (42.2%), and children with higher order of birth (42.4%). About two-fifths of stunted (42.3%) children had SDD. The prevalence of SDD was significantly lower among children of higher educated mothers (14.5%) and higher educated fathers (16.8%) compared to children of parents with no formal education. Similarly, children from the richest quintile had the lowest prevalence of SDD (17.5%) compared to their poorest counterpart (46.9%).

The prevalence of SDD was more than 50% among children of uneducated mothers from poor households (53.4% for poorest and 52.2% for poorer households) (see Additional File, Figure S2). At the subnational level, the highest prevalence of SDD was observed in Karnali Province (45%) followed by Madhesh province (44.2%), and Sudhurpashchim Province (40.1%). (Table 1). Further, when double stratified by provinces and household SES, the highest prevalence of SDD was observed among children from the poorest households in Madhesh province (Fig. 1).

Large gaps in the prevalence of suspected delays in physical and socio-emotional domains were observed between children from the poorest and richest households (see Additional File, Figs. S3, S4). Similarly, wide variations in the prevalence of suspected delays in socio-emotional, learning/cognition, and literacy-numeracy domains were observed between children from the poorest and richest households in Karnali Province (see Additional File; Figs. S4, S5, S6). The prevalence of suspected delay in literacy-numeracy domain was found to be more than 60% among children of mothers with no formal education in all provinces except Gandaki province (see Additional File, Fig. S6).

### Socioeconomic inequality in the prevalence of SDD

The magnitude of absolute socioeconomic inequality in the prevalence of SDD across provinces is presented in Fig. 1, and Table S2. The prevalence of SDD was 32 percentage points higher (SII: −0.32) among children from the poorest households compared to children from the richest households at the national level. Higher levels of pro-poor absolute inequality in the prevalence of SDD was observed among urban children (SII: -0.33). At the subnational level, the highest level of absolute socioeconomic inequality was observed in Lumbini Province (SII = −0.47) followed by, Karnali Province (SII = −0.37), and Bagmati Province (SII = −0.37). In addition, pro-poor inequality was observed in the prevalence of suspected delay in learning/cognition domain among children from rural areas (SII: −0.13) and Lumbini Province (SII: −0.21) (see Additional File, Table S3).

The magnitude of relative socioeconomic inequality in the prevalence of SDD across provinces is presented in Table 2. Children from the richest households in Nepal were 63% less likely (RII: 0.37) to have SDD compared to their poor counterparts. This wealth-based relative inequality was found to be relatively higher in Gandaki Province (RII = 0.57) and Madhesh province (RII = 0.52) (Table 2). Similarly, the concentration curves are above the line of equality indicating that the prevalence of SDD was disproportionately higher among the children from poor households in urban and rural areas (see Additional File, Fig. S7).

**Table 2:** Socioeconomic and maternal education-based inequality in the prevalence of suspected developmental delays among Nepalese children (N = 2870).

	Relative inequality			
	Relative index of inequality (95% CI)	p-value	Concentration index (95% CI)	p-value
Socioeconomic inequality				
National level	0.37 (0.30, 0.44)	** < 0.001**	−0.15 (−0.18, −0.12)	** < 0.001**
Place of residence				
Urban area	0.31 (0.22, 0.39)	** < 0.001**	−0.18 (−0.22, −0.14)	** < 0.001**
Rural area	0.50 (0.37, 0.63)	** < 0.001**	−0.10 (−0.15, −0.06)	** < 0.001**
Province				
Province 1	0.42 (0.15, 0.68)	**0.002**	−0.13 (−0.23, −0.04)	**0.008**
Madhesh province	0.52 (0.33, 0.72)	** < 0.001**	−0.12 (−0.17, −0.06)	** < 0.001**
Bagmati Province	0.21 (0.09, 0.33)	** < 0.001**	−0.24 (−0.32, −0.16)	** < 0.001**
Gandaki Province	0.57 (0.11, 1.03)	**0.015**	−0.11 (−0.24, 0.02)	0.089
Lumbini Province	0.27 (0.15, 0.39)	** < 0.001**	−0.19 (−0.26, −0.13)	** < 0.001**
Karnali Province	0.40 (0.10, 0.70)	**0.009**	−0.13 (−0.20, −0.06)	** < 0.001**
Sudhurpashchim Province	0.45 (0.22, 0.67)	** < 0.001**	−0.10 (−0.18, −0.03)	** < 0.001**
Maternal education-based inequality				
National level	0.33 (0.26, 0.39)	** < 0.001**	−0.15 (−0.18, −0.12)	** < 0.001**
Place of residence				
Urban area	0.28 (0.20, 0.36)	** < 0.001**	−0.17 (−0.21, −0.13)	** < 0.001**
Rural area	0.43 (0.32, 0.54)	** < 0.001**	−0.11 (−0.15, −0.07)	** < 0.001**
Province				
Province 1	0.23 (0.09, 0.37)	**0.002**	−0.22 (−0.31, −0.13)	** < 0.001**
Madhesh province	0.51 (0.31, 0.71)	** < 0.001**	−0.12 (−0.17, −0.06)	** < 0.001**
Bagmati Province	0.25 (0.10, 0.39)	**0.001**	−0.24 (−0.32, −0.16)	** < 0.001**
Gandaki Province	0.39 (0.08, 0.71)	**0.017**	−0.18 (−0.3, −0.07)	**0.002**
Lumbini Province	0.30 (0.16, 0.43)	** < 0.001**	−0.19 (−0.26, −0.13)	** < 0.001**
Karnali Province	0.59 (0.31, 0.87)	** < 0.001**	−0.11 (−0.18, −0.04)	**0.003**
Sudhurpashchim Province	0.71 (0.39, 1.04)	** < 0.001**	−0.06 (−0.13, 0.01)	0.117

*CI* confidence interval.

Significant values are in bold.

### Maternal education-based inequality in the prevalence of SDD

**Figure 2:**
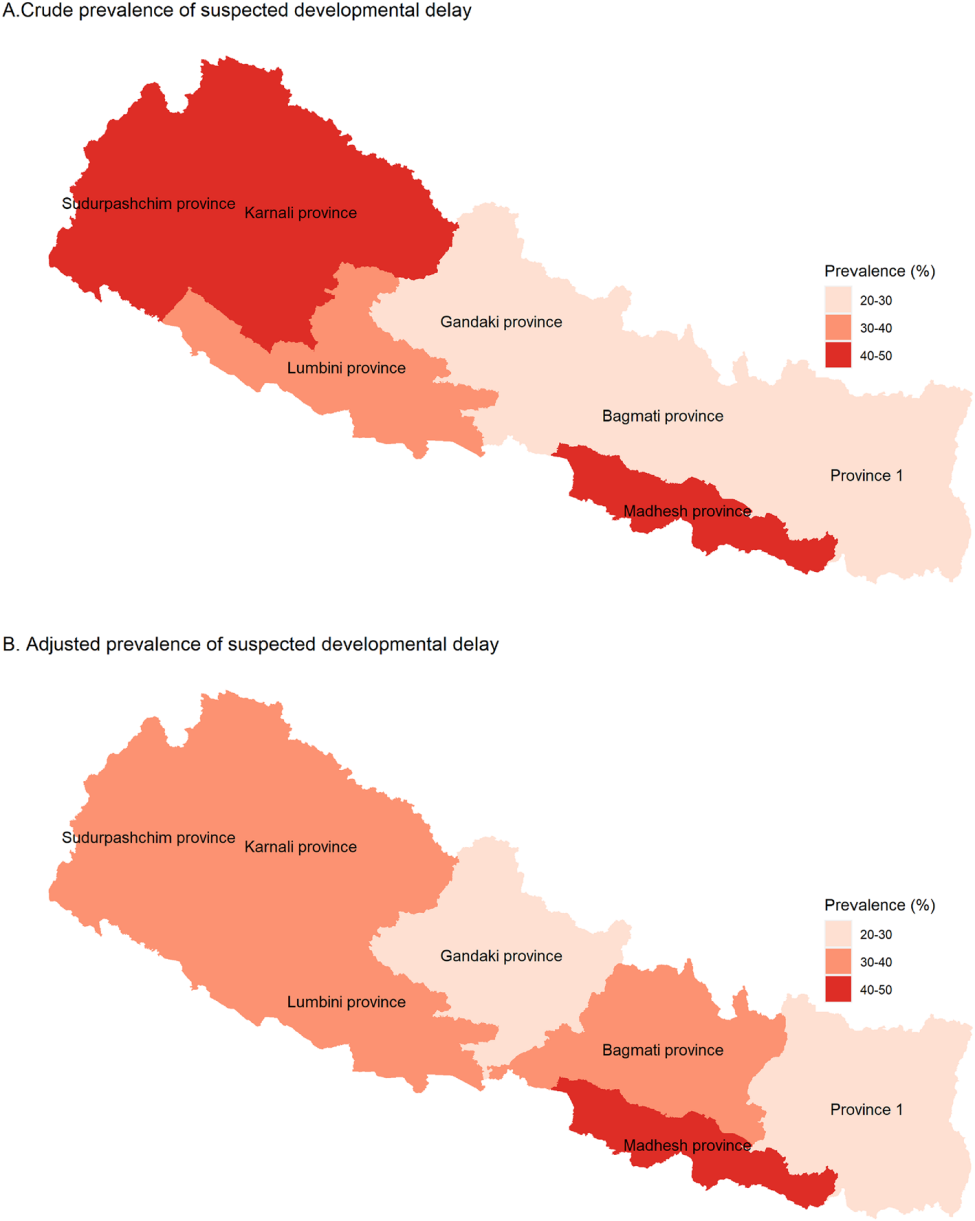
Crude and adjusted prevalence of suspected developmental delays among Nepalese children aged 3–4 years. Models were adjusted for child’s age, sex, nutritional status (stunting), mother’s education, and household socioeconomic status. Exact values along with 95% confidence intervals are presented in Table S5.

The results for maternal education-based absolute inequality in the prevalence of SDD are presented in Fig. 2, and Table S2. The prevalence of SDD was 36 percentage points higher (SII: −0.36) among Nepalese children whose mother had no formal education compared to children of higher educated mothers (Fig. 2; see Additional File, Table S2). Higher level of education-based absolute inequality was observed among urban children (SII: −0.36). At the subnational level, the highest level of education-based absolute inequality was observed in Lumbini Province (SII = −0.44) followed by Bagmati Province (SII: −0.33) and Province 1 (SII: −0.33). In case of delay in literacy-numeracy domain, higher level of education-based absolute inequality was observed among children from Lumbini Province (SII: −0.55) (see Additional File, Table S4).

The results of RII indicated that children of higher educated mothers were 67% less likely (RII: 0.33) to have SDD compared to children whose mothers had no formal education (Table 2). The magnitude of such relative inequality in the prevalence of SDD was found to be highest in Sudhurpashchim Province (RII = 0.71), followed by Karnali Province (RII = 0.59) (Table 2).

### Risk factors for SDD

Table 3 presents the crude and adjusted ORs for SDD, obtained from multilevel logistic regression models. In the fully adjusted model, children aged 48–59 months were 81% less likely to have SDD (aOR = 0.19; 95% CI = 0.13–0.29) compared to children aged 36–47 months. Stunted children, who manifests a physical manifestation of chronic malnutrition and has been linked to higher rates of suboptimal development^40^, had 1.78 times higher odds of having SDD (aOR = 1 0.78; 95% CI = 1.22–2.58) compared to those who are not stunted. The odds of SDD were significantly lower among children of secondary and higher educated mothers (aOR = 0.30, 95% CI = 0.17–0.52 for secondary educated mother and aOR = 0.12, 95%CI = 0.05–0.31 for higher educated mother) compared to children of uneducated mothers. Similarly, children of rich households had lower odds of SDD. Children from Madhesh province (aOR = 6.37, 95% CI = 3.03–13.37), Lumbini Province (aOR = 4.01, 95% CI = 1.96–8.21) and Sudhurpashchim Province (aOR = 2.57, 95% CI = 1.19–5.53) had higher odds of having SDD compared to those from Province 1.

**Table 3:** Risk factors of suspected developmental delays among Nepalese children (N = 2870).

Variables	Categories	Odds ratio (95% confidence interval)				
		Model 1^a^	Model 2^b^	Model 3^c^	Model 4^d^	Model 5^e^
Child characteristics						
Age of children	36–47 months (ref.)	1.00	1.00	1.00	1.00	1.00
	48–59 months	0.51 (0.41–0.64)***	0.22 (0.15–0.32)***	0.20 (0.14–0.30)***	0.19 (0.13–0.29)***	0.19 (0.13–0.29)***
Sex of children	Boys (ref.)	1.00	1.00	1.00	1.00	1.00
	Girls	0.96 (0.80–1.17)	0.88 (0.63–1.23)	0.90 (0.64–1.26)	0.90 (0.64–1.27)	0.88 (0.62–1.24)
Birth order	1 (ref.)	1.00	1.00	1.00	1.00	1.00
	2–4	1.88 (1.41–2.51)***	2.60 (1.70–3.96)***	1.76 (1.14–2.73)**	1.56 (0.99–2.46)	1.41 (0.89–2.22)
	≥ 5	2.24 (1.44–3.50)***	3.83 (1.89–7.76)***	1.98 (0.87–4.49)	1.55 (0.67–3.58)	1.20 (0.52–2.79)
Stunting	No (ref.)	1.00	1.00	1.00	1.00	1.00
	Yes	1.69 (1.38–2.08)***	2.43 (1.69–3.51)***	2.08 (1.44–2.99)***	1.93 (1.33–2.81)**	1.78 (1.22–2.58)**
Parent’s characteristics						
Mother’s age	15–24 years (ref.)	1.00		1.00	1.00	1.00
	25–34 years	1.11 (0.88–1.40)		1.14 (0.75–1.73)	1.35 (0.87–2.08)	1.54 (0.99–2.38)
	35 years and above	1.22 (0.87–1.69)		0.60 (0.32–1.13)	0.78 (0.41–1.49)	1.07 (0.56–2.06)
Mother’s education	No education (ref.)	1.00		1.00	1.00	1.00
	Basic education	0.58 (0.45–0.75)***		0.44 (0.28–0.70)***	0.51 (0.32–0.83)**	0.67 (0.41–1.08)
	Secondary education	0.34 (0.27–0.44)***		0.16 (0.09–0.27)***	0.22 (0.13–0.38)***	0.30 (0.17–0.52)***
	Higher education	0.18 (0.10–0.30)***		0.05 (0.02–0.12)***	0.09 (0.03–0.26)***	0.12 (0.05–0.34)***
Household-level characteristics						
Household socio-economic status	Poorest (ref.)	1.00			1.00	1.00
	Poorer	0.75 (0.55–1.04)			0.56 (0.32–0.97)*	0.47 (0.26–0.85)*
	Middle class	0.56 (0.42–0.75)***			0.44 (0.25–0.77)**	0.33 (0.18–0.62)**
	Richer	0.53 (0.37–0.76)***			0.53 (0.29–0.96)*	0.41 (0.21–0.79)**
	Richest	0.24 (0.16–0.37)***			0.15 (0.07–0.33)***	0.13 (0.05–0.31)***
Household size	1–4 persons (ref.)	1.00			1.00	1.00
	5–6 persons	0.93 (0.74–1.15)			0.89 (0.59–1.34)	0.87 (0.58–1.32)
	> 6 persons	1.11 (0.82–1.49)			1.07 (0.65–1.79)	1.08 (0.64–1.80)
Iodine level in the salt consumed	None (0 ppm) (ref.)	1.00			1.00	1.00
	Inadequate (< 15 ppm)	1.18 (0.72–1.94)			0.93 (0.37–2.33)	0.96 (0.38–2.42)
	Adequate (≥ 15 ppm)	1.11 (0.70–1.74)			0.86 (0.37–1.99)	1.00 (0.43–2.32)
Community-level characteristics						
Place of residence	Urban area (ref.)	1.00				1.00
	Rural area	1.41 (1.12–1.75)**				1.27 (0.83–1.94)
Province	Province 1 (ref.)	1.00				1.00
	Madhesh province	2.64 (1.75–3.98)***				6.37 (3.03–13.37)***
	Bagmati Province	1.18 (0.78–1.78)				2.24 (1.07–4.67)*
	Gandaki Province	0.86 (0.54–1.37)				1.01 (0.43–2.39)
	Lumbini Province	2.22 (1.44–3.42)***				4.01 (1.96–8.21)***
	Karnali Province	2.72 (1.72–4.30)***				2.34 (0.99–5.53)
	Sudhurpashchim Province	2.23 (1.45–3.37)***				2.57 (1.19–5.56)***
Random-effects parameters						
Community level	Variance		1.66	1.12	1.03	0.75
	ICC		0.15	0.10	0.10	0.07
Household level	Variance		6.23	6.39	6.43	6.50
	ICC		0.70	0.70	0.69	0.69
Model comparison						
Likelihood ratio test^f^	Chi-square statistic		314.86	289.15	277.51	257.21
	P value		< 0.001	< 0.001	< 0.001	< 0.001
AIC			3097	3026	2854	2831
BIC			3144	3103	2977	2996

*ref.*  reference category, *ppm* parts per million, *SD* standard deviation, *SE* standard error.

***p < 0.001, **p < 0.01, *p < 0.05.

^a^Model 1: crude models.

^b^Model 2: only child characteristics were included in the model.

^c^Model 3: additionally adjusted for parental characteristics.

^d^Model 4: additionally adjusted for household related characteristics.

^e^Model 5: additionally adjusted for community characteristics.

^f^Based on the results on likelihood ratio tests, estimates of multilevel logistic regression models were preferred than fixed effect models.

### Risk factors adjusted prevalence of SDD

The risk factor adjusted prevalence of SDD at the subnational level is presented in Fig. 2 and Table S5. The crude prevalence of SDD was found to be highest in Karnali Provinces (45.0%, 37.4–52.8), followed by Madhesh province (44.2%, 38.2–50.3), whereas the risk factor adjusted prevalence of SDD was found to be highest in Madhesh province (42.3%, 38.1–46.5), followed by Lumbini Province (37.5%, 33.1–41.9) (Fig. 2).

## Discussion

This is the first comprehensive study to investigate the regional variation in the magnitude of inequality in the prevalence of SDD among Nepalese children using a nationally representative survey. The findings of this study showed that more than one-third of Nepalese children experience SDD, with relatively higher prevalence in rural areas and in several provinces including Karnali Province, Madhesh province, and Sudhurpashchim Province. Prevalence was significantly higher among children from poor households and those born to lower educated mothers. Wide inequalities in the prevalence of SDD, in terms of SES and mother’s education, as well as subnational variation in the magnitude of such inequalities were observed. In addition, children being stunted, lower levels of mother’s education and disadvantaged household socioeconomic status were identified as significant risk factors for SDD.

The findings of the present study highlighted that the national prevalence of SDD among children aged 3–4 years in Nepal has reduced just a little during 2014–2019 (from 35.6% in 2014 to 34.8% by 2019)^41^. A previous study also mentioned similar level of prevalence (38%) in South Asia^7^. Despite global efforts to foster child development, the prevalence of SDD in many LMICs is still relatively high^8,9^ and our study shows Nepal is not an exception. Although the prevalence of SDD in Nepal as observed in the present study is lower than the overall prevalence for LICs (41.2%), as reported in the previous study^8^, it indicates higher burden of developmental delays in Nepal. When investigated by domains, the prevalence of suspected delay was relatively higher for literacy-numeracy domain and socio-emotional domain. The prevalence of suspected delay in socioemotional domain (44%) in Nepal, as found in the present study, is even higher than the average estimates for South Asian region (31.0%)^8^. The higher burden of SDD among Nepalese children could be due to the shortcomings in the ongoing interventions for childhood development^42^, as well as lack of parental recognition of developmental milestones for their children^43^. Primary education in Nepal usually begins at the age of 5 or 6 years, and sending children to preschool is not yet common especially among people of lower socio-economic background. Additionally, the early childhood centers are under-resourced and without trained early childhood development teachers, particularly in rural settings, they are often just a place for children to spend time while their parents are working. Hence, the higher prevalence of SDD in the literacy-numeracy domain may reflect lack of opportunity for training during the early years of schooling. The lower prevalence of SDD in children aged 48–59 months (27.0%) compared to their younger counterparts (42.2%), as well as those not attending early childhood education (54.9%) may explain the effect of delayed schooling on the higher prevalence of SDD in the literacy-numeracy domain. Our results show the highest prevalence of SDD in the literacy-numeracy domain compared to other domains, which may have significantly contributed to increasing the overall prevalence of SDD. The very low prevalence of SDD in the physical and learning/cognition domain and the higher prevalence in the other two domains may reflect the limitation of the ECDI in capturing developmental milestones, as criticized by McCoy et al.^7^.

At the subnational level, Karnali Province exhibited the highest prevalence of SDD (45%) followed by Madhesh province (44.2%), and Sudhurpashchim Province (40.1%), which are even higher than the national prevalence. On the other hand, this study found Gandaki Province had the lowest prevalence of SDD (20.5%) followed by Province 1 (23.1%). Such regional variation was observed even after adjusting for potential risk factors of SDD, which highlights the need for province-specific tailored interventions for reduction of the prevalence. The possible reasons for this subnational variation in the prevalence of SDD in Nepal might be the provincial disparities in level of economic development as well as poverty, literacy, culture, and land topography. It should be noted that Karnali Province, Sudhurpashchim Province and Madhesh province have relatively lower levels of development, in terms of human development index, and higher levels of poverty compared to the national average^44–46^. Moreover, female literacy rates in those provinces are also lower than the national female literacy rate^45^. Similarly, the total fertility rate of 3.0 child per women in Madhesh province is higher than the national average of 2.3^47^. The higher number of children in a low socioeconomic background may lead to less attention given to each child, thus affecting parental recognition of the milestone, or hindering the child’s development as a whole.

In line with a previous study conducted in 63 LMICs^8^, the present study observed major gaps in the prevalence of suspected delays in physical and socio-emotional domains between children from the poorest and richest households. The magnitude of socioeconomic inequality in the prevalence of SDD at the national level in the present study, in terms of absolute inequality, was found to be higher than the magnitude of inequality reported in the previous study conducted in Nepal^8^. That previous study, which used survey data collected in 2014, reported about 23 percentage points higher prevalence of SDD among children from the poorest households compared to the richest households, whereas this study reported 32% percentage points higher prevalence, indicating a sharp increase in the magnitude of inequality^8^. Such wealth-based inequality in SDD was even higher among children from urban areas than rural children. We could not compare this finding with any previous studies due to lack of similar studies, but the prevalence of SDD was reported to be higher among children from rural areas compared to urban children, which is consistent with previous studies from Nepal and other LMICs^7,8^. Disadvantaged households’ economic condition, particularly poverty, is associated with higher risk of developmental delays among children as those children are likely to experience unfavorable household conditions such as inadequate home stimulating environment and unavailability of learning material like toys^48^. lack of childcare, inadequate nutrition, exposure to indoor air pollution, violence etc^15^. Moreover, the magnitude of wealth-based inequality in SDD varies across provinces with the widest gaps in the prevalence of SDD observed in Madhesh province, highlighting the urgent need to introduce appropriate policies and early childhood development programs aimed at educating and empowering women to reduce the burden of SDD in this province.

The prevalence of SDD was found to be lower among children of higher educated mothers, which is consistent across provinces. Previous studies conducted in LMICs also reported similar findings^8,14^. Similar to socioeconomic inequality, the study also revealed that the magnitude of education-based inequality in SDD varies across provinces. A recent Chinese study observed significant variation in the magnitude of maternal education-based inequality in child development across regions^14^. That study noted higher levels of education-based inequality in the least-developed regions compared to developed regions^14^, which is in line with our study. The most important contribution of our study to the existing literature is the subnational assessment of inequalities in SDD, which would help policy makers to formulate appropriate policies. Given the strong effect of maternal education on childhood development identified in this study, and a larger proportion of women compared to men with no education provided, the Nepalese government should redouble efforts to improve education of girls and young women, and should also consider moves to enhance adult education for the larger number of young women who are entering childbearing age without adequate formal education. More work is also needed internationally on strategies to assist parents with low education levels in supporting their children’s development, to ensure that their own inadequate educational opportunities do not create an additional burden in the development of their children.

Our study identified being stunted, along with household SES and mother’s education, as a significant risk factor for SDD among children, which is also supported by previous studies^7,21,49–52^. A recent study mentioned that around 10% of the prevalence of suspected delay in cognitive domain could be eliminated by eliminating stunting in LMICs, highlighting the need for reducing stunting prevalence in relation to child development^53^. Previous studies also found that, elimination of poverty, and childhood stunting, ensuring home stimulation for each children, at least secondary-level of education for each mother and improved sanitation during their early childhood can potentially reduce approximately 80% prevalence of the cognitive delay^53^. Therefore, prevention of childhood stunting, while focusing on reduction of poverty and improving the literacy rate among mother might be beneficial for minimizing the burden of developmental delays among Nepalese children. In addition to basic activities to eliminate extreme poverty, a simple and cost-effective intervention to improve childhood nutrition and reduce stunting is action to enhance breastfeeding^54^.

The major strength of this study includes nationally representative sample. This is the first study that quantified both socioeconomic and education-based inequality in SDD among Nepalese children at the subnational level. Moreover, with the application of SII, RII and concentration index, we evaluated the level of both absolute and relative inequality. Along with these strengths, the study has a few limitations which include its cross-sectional design and small sample size. Despite the small sample size, the outcome of interest was not rare and differences in the prevalence of outcome between groups of interest were large, indicating a large effect size that would be easily detected by moderately-sized studies. Although calculation of power is complicated in multiple logistic regression of cluster-sampled surveys, we are confident that our study is not underpowered given these characteristics^55–57^. Due to the lack of information, we could not adjust for several important variables including maternal nutrition, early exposure to environmental factors, or genetic factors. In addition, the assessment of the outcome might be subject to social desirability bias as mothers/caregivers in LMICs are often reluctant to report poor development of their children because of the fear of social stigma^58^. Finally, the ECDI was calculated based on the 10-item instrument, which is not a time-dependent instrument although it is widely used in LMICs and recognized by UNICEF^1^. However, despite limitations, the ECDI is able to identify children with SDD and facilitate comparison between different population groups. Application of other neurodevelopment instruments such as the Ages and Stages Questionnaires might provide better understanding to track the timely development of children.

## Conclusion

Despite continual efforts to foster child development, one in three children in Nepal experience SDD. The prevalence was found to be relatively higher among children from rural areas and those from Madhesh province, Lumbini Province, Karnali Province and Sudhurpashchim Province. Wide socioeconomic and education-based inequality in SDD were observed and the magnitude of such inequalities vary across provinces. Lumbini Province exhibited the highest magnitude of socioeconomic and education-based inequality. Thus, province-specific tailored interventions should be designed for promoting early childhood development, and children from marginalized and hard-to-reach communities must be prioritized in national health policies and programs. Cost-effective early childhood development interventions could be incorporated with existing maternal and child health and nutrition programs. In addition, multisectoral efforts should be scaled up to address the potential factors that directly or indirectly affects early childhood development such as increasing female literacy rate and reduction of poverty and stunting, which may help to reduce the burden and existing inequalities in developmental delays and to achieve the SDG target 4.2 in Nepal.

## Supplementary Information

Supplementary Information.

## Data Availability

This secondary analysis of the current study is based on publicly available MICS datasets and permission was granted for use upon the request from UNICEF/MICS website (http://mics.unicef.org/surveys).
