# Supplementary material for: Socioeconomic and education-based inequality in suspected developmental delays among Nepalese children: a subnational level assessment
Source: Sci Rep. 2023 Mar 23;13:4750. doi: 10.1038/s41598-023-31629-1 (PMC10036624; doi:10.1038/s41598-023-31629-1)
Supplement: Supplementary file 1 — Supplementary Information. [file 41598_2023_31629_MOESM1_ESM.docx]

**Supplementary information**

**Socioeconomic and education-based inequality in suspected developmental delays among Nepalese children: a subnational level assessment**

Kiran Acharya, MPH^1^[^†^](https://www.thelancet.com/journals/lancet/article/PIIS0140-6736(18)32558-3/fulltext), Md. Shafiur Rahman, PhD^2,3^[^†^](https://www.thelancet.com/journals/lancet/article/PIIS0140-6736(18)32558-3/fulltext)^*^; Md. Rashedul Islam, PhD^4,5^; Stuart Gilmour, PhD^6^; Bibha Dhungel, MPH^6,7^; Rajendra P Parajuli, PhD^8^; Tomoko Nishimura, PhD^2,3^; Atsushi Senju, PhD^2,3^; Kenji J. Tsuchiya, MD, PhD^2,3^

^1^New ERA, Rudramati Marg, Kalopul, Kathmandu, Nepal

^2^Research Centre for Child Mental Development, Hamamatsu University School of Medicine, Hamamatsu, Japan

^3^United Graduate School of Child Development, Osaka University, Kanazawa University, Hamamatsu University School of Medicine, Chiba University and University of Fukui, Hamamatsu, Japan

^4^Department of Global Health Policy, The University of Tokyo, Tokyo, Japan

^5^National Cancer Center Institute for Cancer Control, Tokyo, Japan

^6^Graduate School of Public Health, St. Luke's International University, Tokyo, Japan

^7^Department of Health Policy, National Centre for Child Health and Development, Tokyo, Japan

^8^Central Department of Zoology, Central Campus, Institute of Science & Technology (IOST), Tribhuvan University, Kritipur‑1, Kathmandu, Nepal

[^†^](https://www.thelancet.com/journals/lancet/article/PIIS0140-6736(18)32558-3/fulltext)These authors share first authorship

***Corresponding author**

Md Shafiur Rahman, PhD

Senior Assistant Professor, Research Centre for Child Mental Development

Hamamatsu University School of Medicine

1-20-1 Handayama, Higashi-Ku, Hamamatsu 431-3192, Japan

Tel: +81 53435 2331; Fax: +81 53435 2291

E-mail: srahman@hama-med.ac.jp

**Supplementary information**

**Additional File: Method S1-S2**

**Method S1**: Items for assessing early childhood development

**Method S2**: Assessment of household socioeconomic status

**Additional File: Figure S1-S7**

**Figure S1**: Participant selection.

**Figure S2**: Prevalence of suspected developmental delays among Nepalese children aged 3-4 years by household socioeconomic status and maternal education.

**Figure S3**: Prevalence of suspected delay in physical domain among Nepalese children aged 3-4 years by household socioeconomic status and maternal education.

**Figure S4**: Prevalence of suspected delay in socio-emotional domain among Nepalese children aged 3-4 years by household socioeconomic status and maternal education.

**Figure S5**: Prevalence of suspected delay in learning/cognition domain among Nepalese children aged 3-4 years by household socioeconomic status and maternal education.

**Figure S6**: Prevalence of suspected delay in literacy-numeracy domain among Nepalese children aged 3-4 years by household socioeconomic status and maternal education.

**Figure S7**: Concentration curves of proportion of suspected developmental delays among Nepalese children.

**Tables S1-S2**

**Table S1:** Prevalence of suspected delays in physical, socio-emotional, learning/cognition, literacy-numeracy domains among Nepalese children aged 3-4 years (N=2,870).

**Table S2:** Socioeconomic and education-based absolute inequality in the prevalence of suspected developmental delays among Nepalese children aged 3-4 years (N=2,870).

**Table S3:** Socioeconomic inequality in the prevalence of suspected delays in physical, social-emotional, learning/cognition, and literacy-numeracy domains among Nepalese children aged 3-4 years (N=2,870).

**Table S4:** Maternal education-based inequality in the prevalence of suspected delays in physical, social-emotional, learning/cognition, and literacy-numeracy domains among Nepalese children aged 3-4 years (N=2,870).

**Table S5**: Variations in the prevalence of suspected developmental delays among Nepalese children before and after adjusting for risk factors (N=2,870).

**Supplemental Method**

**Method S1: Items for assessing early childhood development**

The following 10 items were used in Nepal MICS 2019 to construct Early childhood development index (ECDI). Detailed of each item are presented elsewhere [1, 2].

| **Domain** | **Decisions** | **Item/question** | **Response** |
| --- | --- | --- | --- |
| Learning-cognition domain  (2 items) | A child is considered developmentally on track for learning/cognition domain if at least one item is on track | Whether the child follow simple directions on how to do something correctly? | Yes, no |
|  |  | When given something to do, whether the child is able to do it independently? | Yes, no |
| Socio-emotional domain  (3 items) | A child is considered developmentally on track for socio-emotional domain if at least two items are on track | Whether the child get along well with other children? | Yes, no |
|  |  | Whether the child kick, bite, or hit other children or adults? | Yes, no |
|  |  | Whether the child get distracted easily? | Yes, no |
| Literacy-numeracy domain  (3 items) | A child is considered developmentally on track for literacy/-numeracy domain if at least two items are on track | Whether the child can identify or name at least ten letters of alphabet? | Yes, no |
|  |  | Whether the child can read at least four simple, popular words? | Yes, no |
|  |  | Whether the child know the name and recognize the symbol of all numbers from 1 to 10? | Yes, no |
| Physical domain  (2 items) | A child is considered developmentally on track for physical domain if at least one item is on track | Whether the child pick up a small object with two fingers, like a stick or a rock, from the ground? | Yes, no |
|  |  | Whether the child sometimes too sick to play? | Yes, no |

**Method S2: Assessment of household socioeconomic status**

Household socioeconomic status (SES) was assessed using a wealth index proxy variable. Given the difficulty of collecting income and expenditure data in developing countries, many surveys including MICS collect data on characteristics that relate to economic status such as household ownership of consumer goods, dwelling materials, sources of drinking water, types of sanitation facilities, etc. The household wealth score is then calculated, using a principal component analysis, based on information on household characteristics and assets. Then, the entire sample is ranked according to the wealth score and divided into quintiles, where the first quintile (Q1) presents the poorest 20% of the household population and vice-versa. The wealth score of NMICS 2019 was calculated by the survey authority and the details of the calculation procedure for wealth index can be found elsewhere [2-4].

**Figure S1**: Participant selection

91 mother-child dyads did not complete the survey

3,760 children aged 0-2 years were excluded due to out of scope of the current study

2,898 children aged 3-4 years were selected for the current study

6,658 of the children were participated in the study

7,250 households

5,550 households

290 urban EAs

222 Rural EAs

Out of 36,020 total enumeration areas (EAs) in 2011 Nepal Census, 512 EAs were selected

12,800 households selected

12,655 households interviewed

6,749 children under age five listed in the household

12,687 households occupied


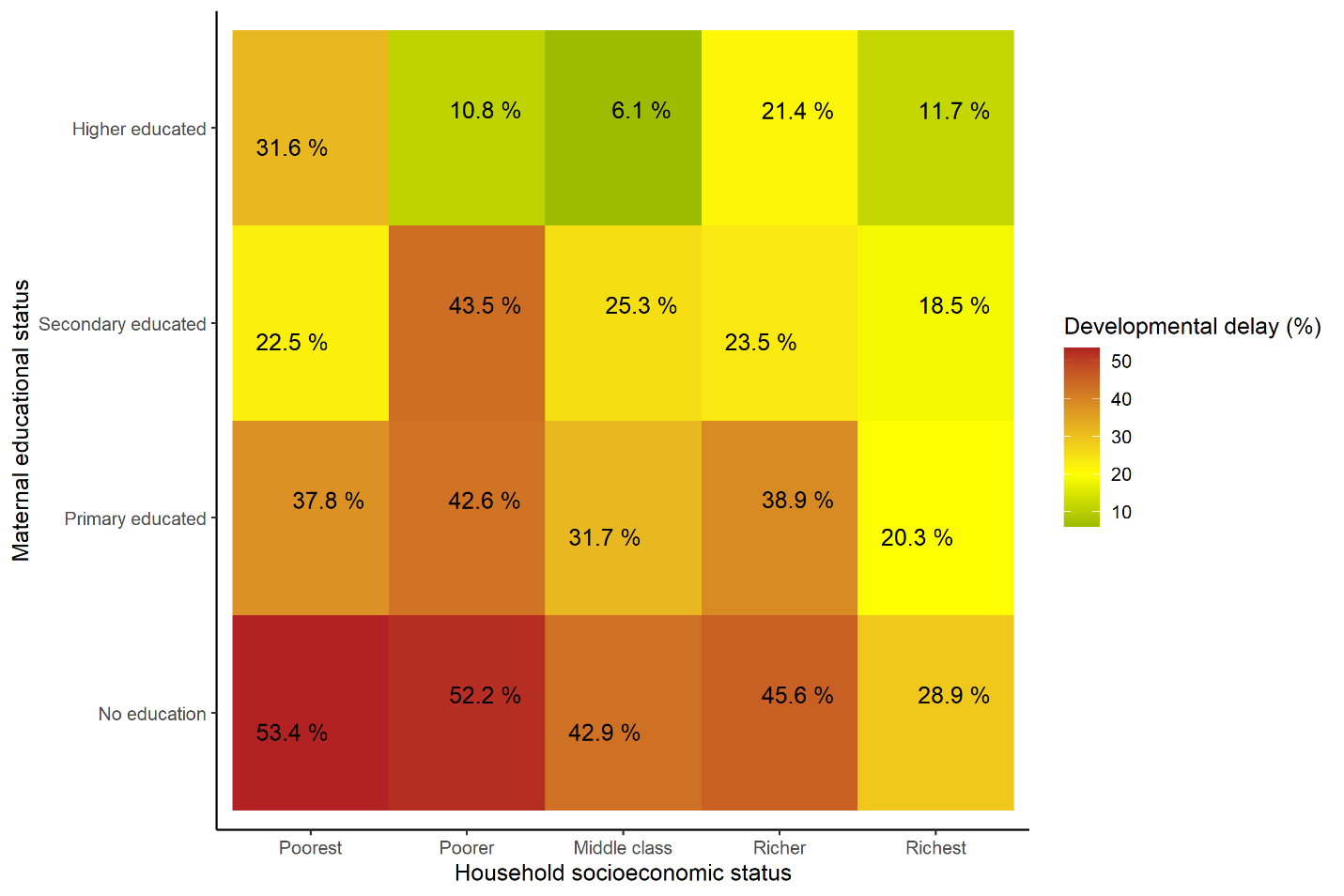


**Figure S2**: Prevalence of suspected developmental delays among Nepalese children aged 3-4 years by household socioeconomic status and maternal education


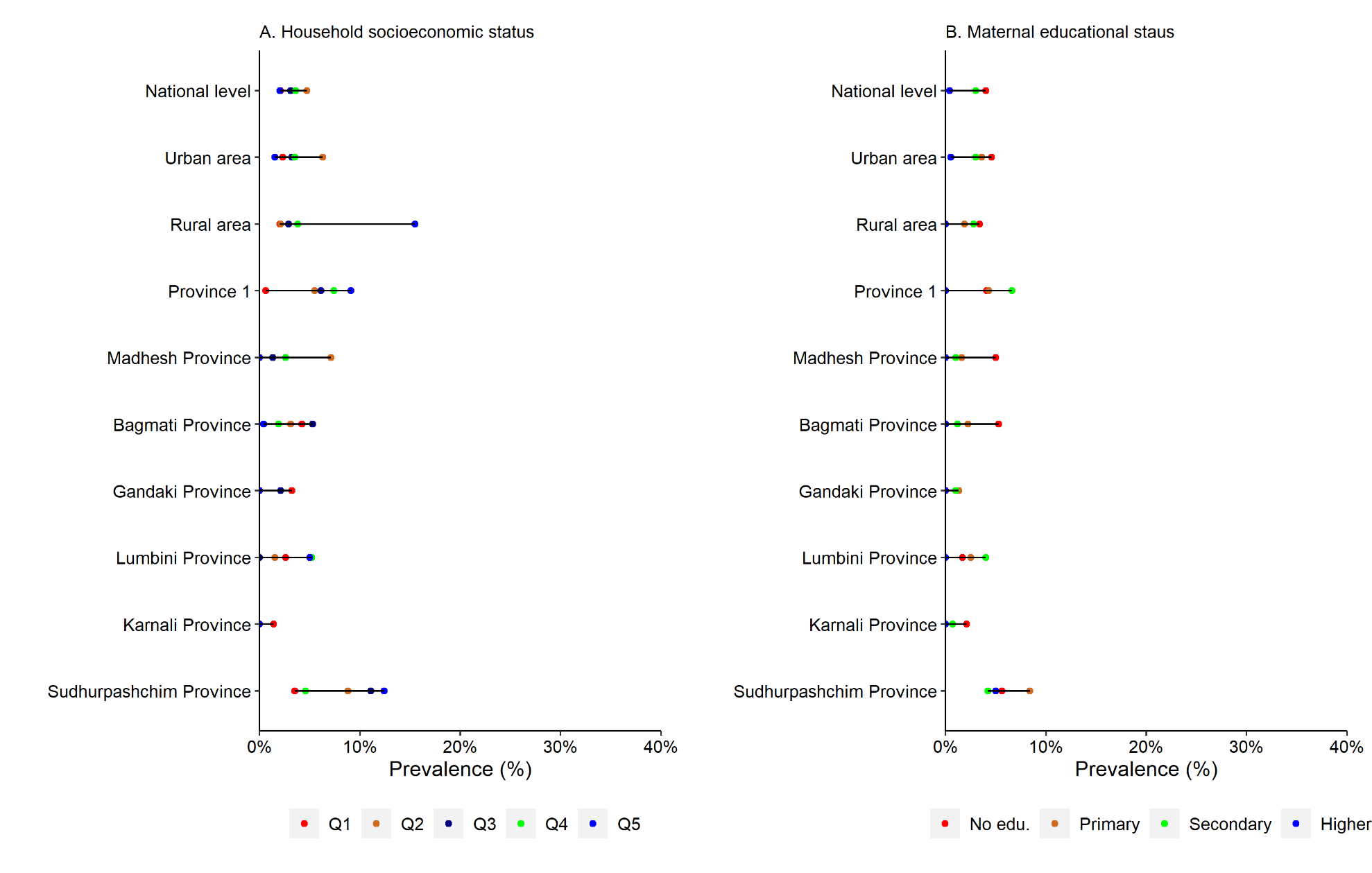


**Figure S3**: Prevalence of suspected delay in physical domain among Nepalese children aged 3-4 years by household socioeconomic status and maternal education


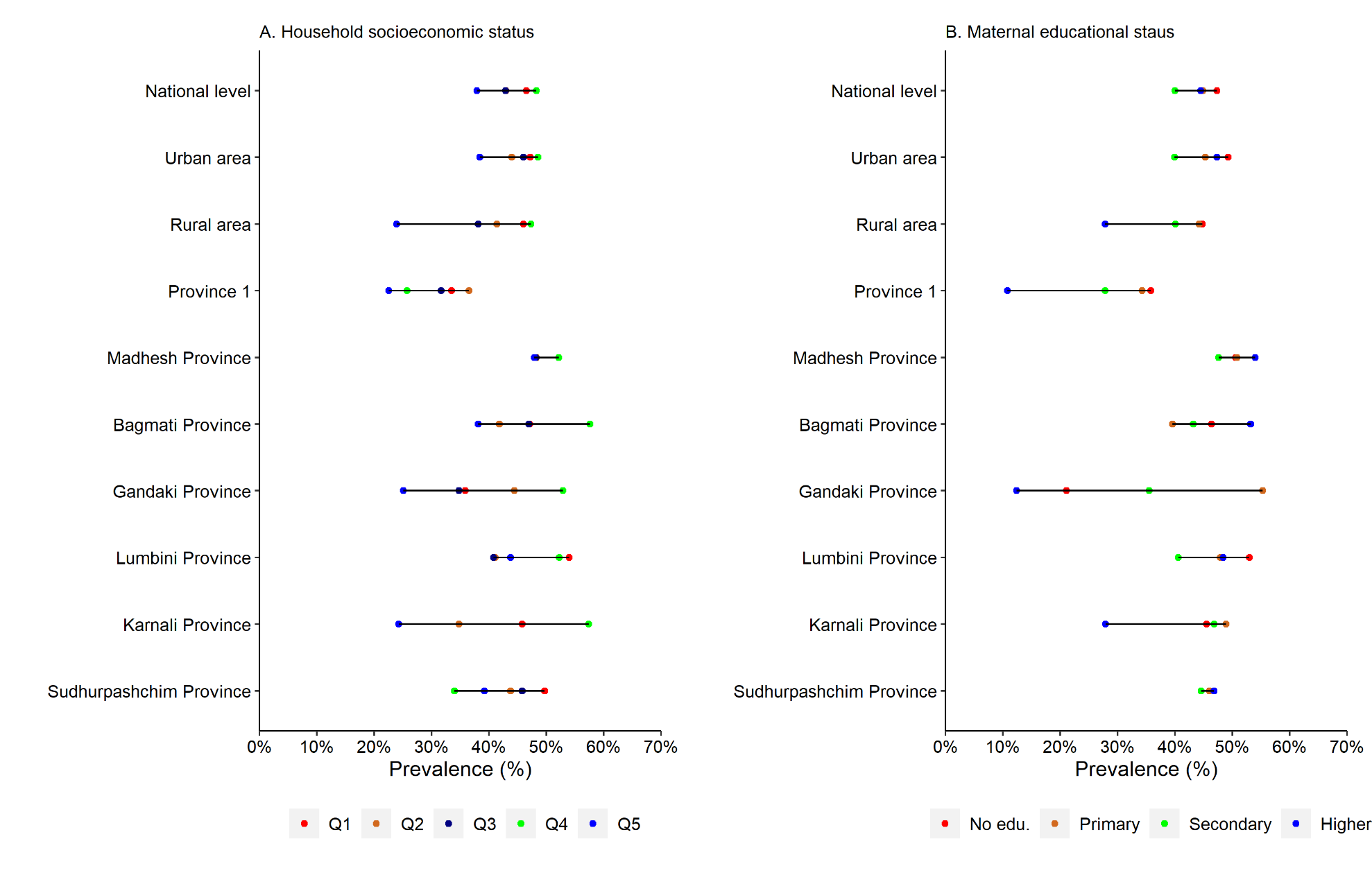


**Figure S4**: Prevalence of suspected delay in socio-emotional domain among Nepalese children aged 3-4 years by household socioeconomic status and maternal education


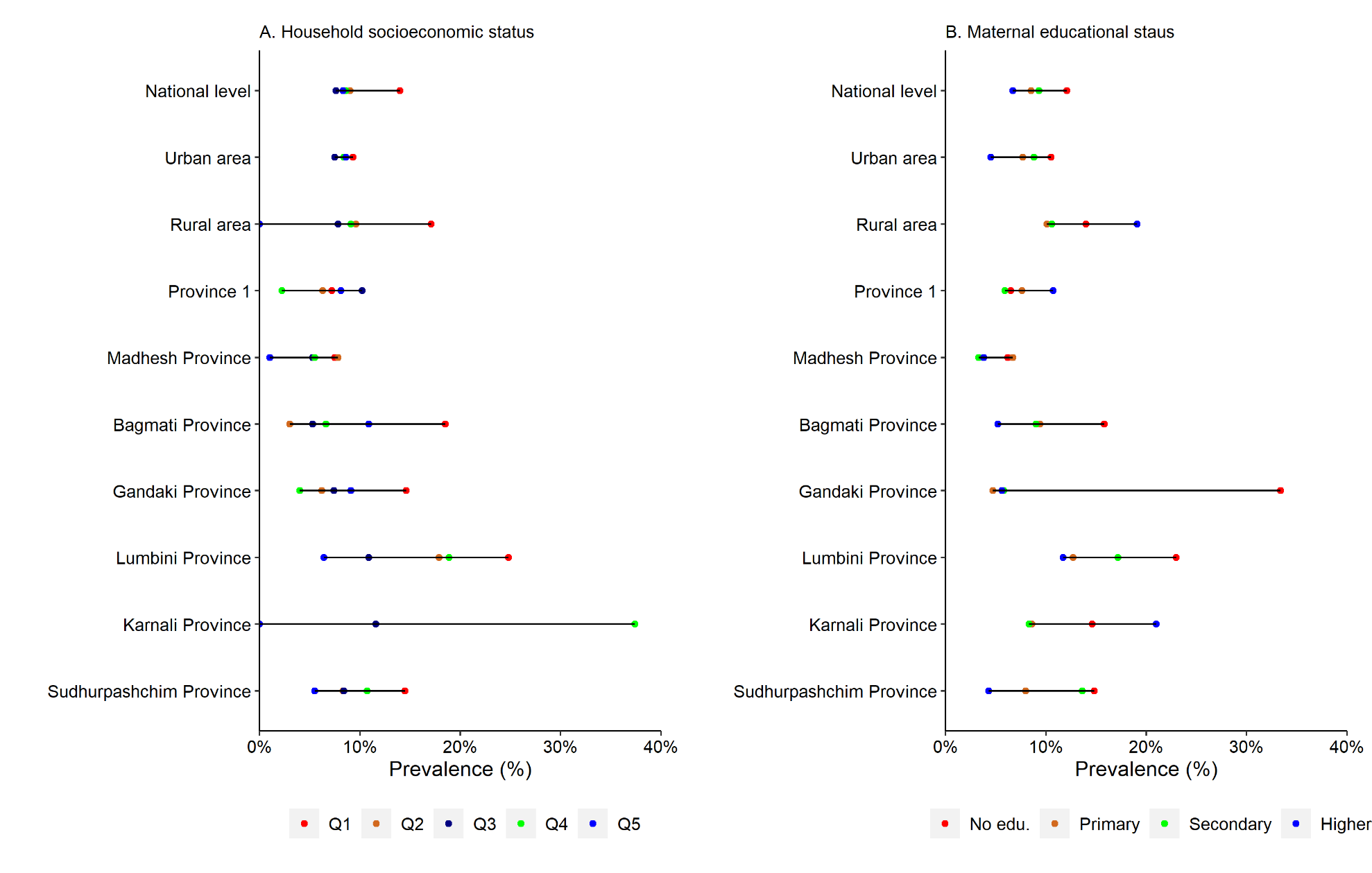


**Figure S5**: Prevalence of suspected delay in learning/cognition domain among Nepalese children aged 3-4 years by household socioeconomic status and maternal education


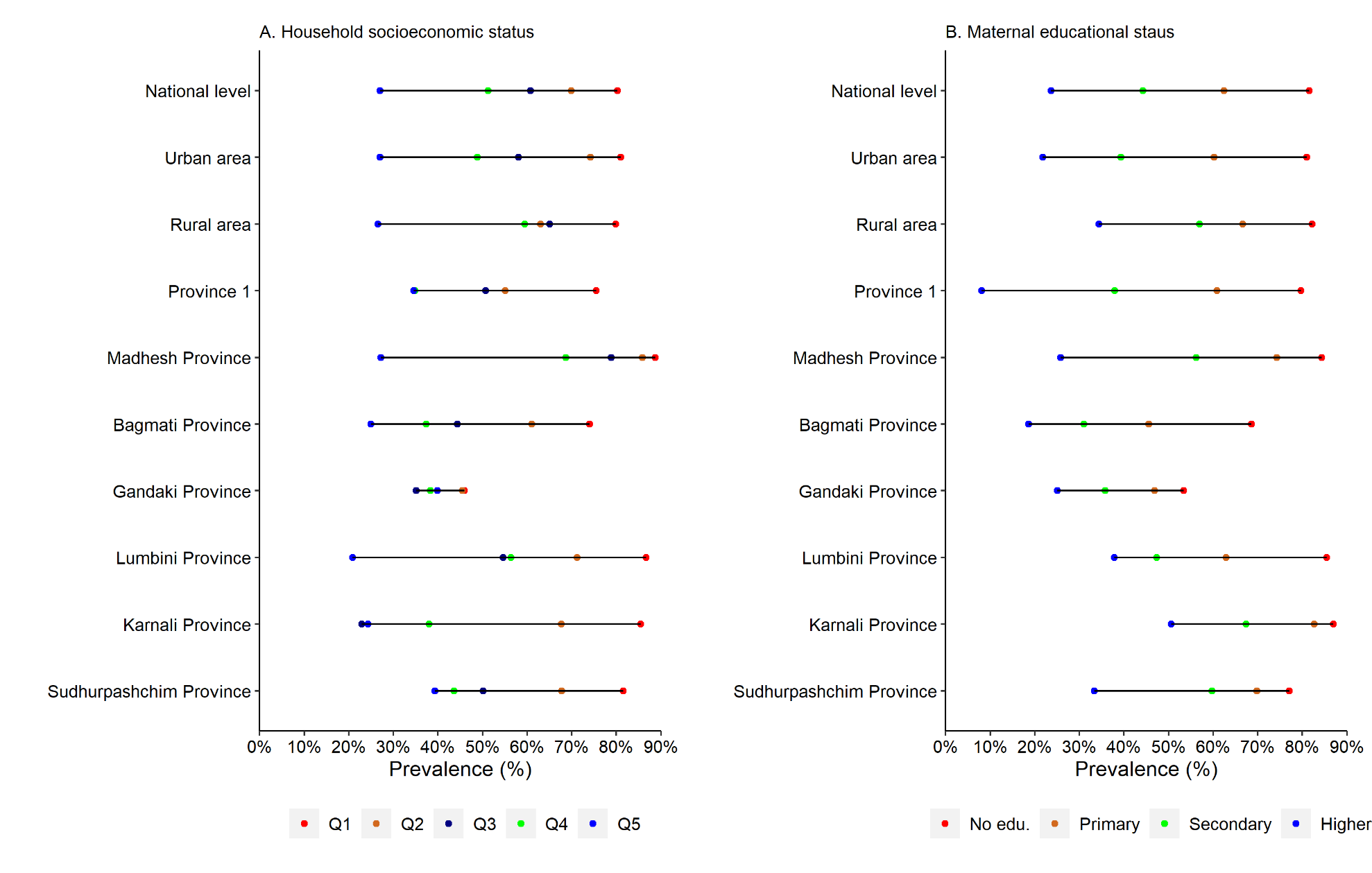


**Figure S6**: Prevalence of suspected delay in literacy-numeracy domain among Nepalese children aged 3-4 years by household socioeconomic status and maternal education


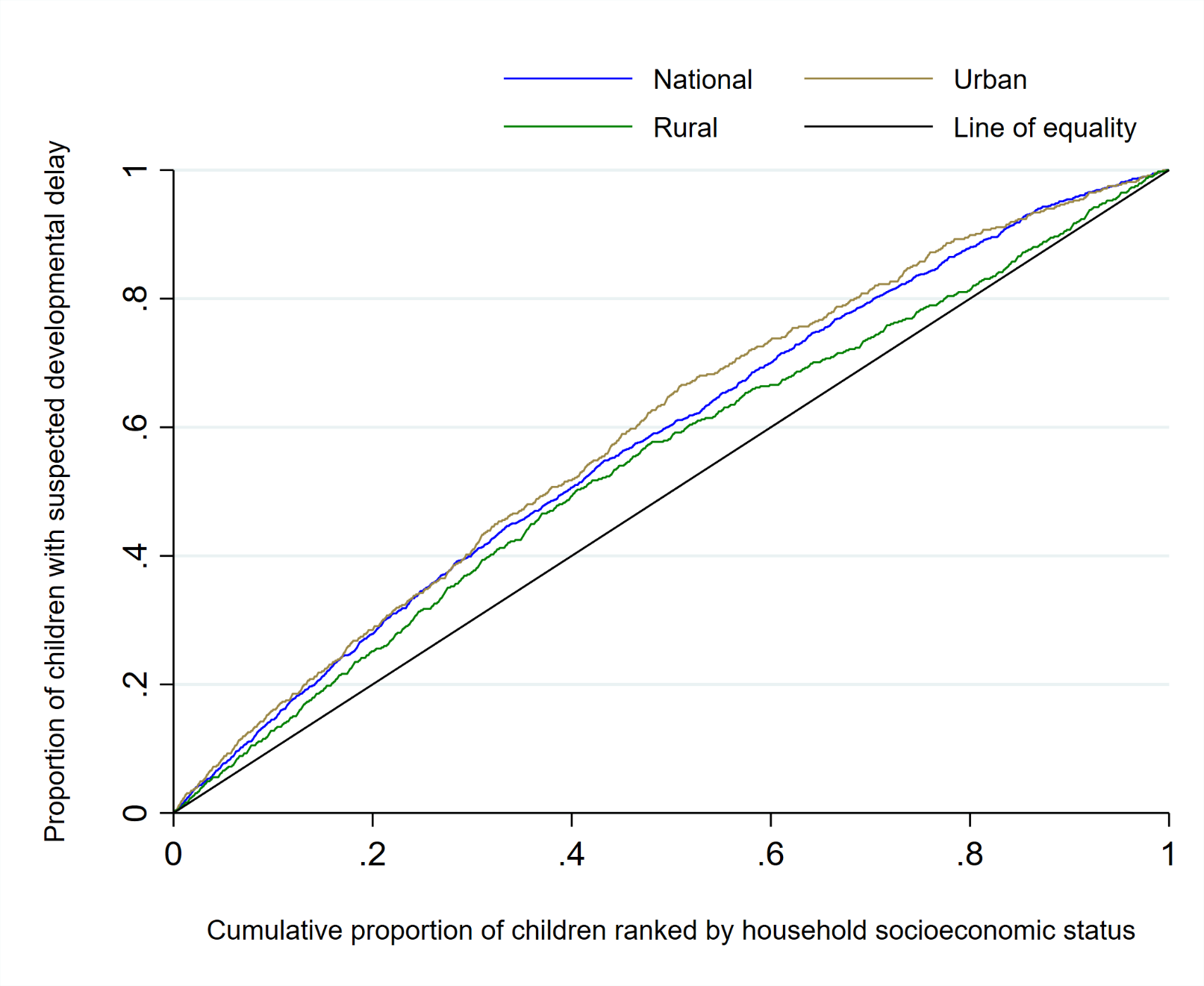


**Figure S7**: Concentration curves of proportion of suspected developmental delays among Nepalese children

**Table S1:** Prevalence of suspected delays in physical, socio-emotional, learning/cognition, literacy-numeracy domains among Nepalese children aged 3-4 years (N=2,870)

|  | **Prevalence (95% confidence interval)** | | | |
| --- | --- | --- | --- | --- |
|  | Physical domain | Social-emotional domain | Learning/cognition domain | literacy-numeracy domain |
| **National level** | 3.1 (2.4–4.0) | 44.0 (41.3–46.7) | 9.7 (8.1–11.4) | 59.6 (56.5–62.6) |
| **Place of residence** |  |  |  |  |
| Urban area | 3.3 (2.4–4.6) | 44.5 (40.9–48.3) | 8.4 (6.5–10.9) | 54.5 (50.3–58.7) |
| Rural area | 2.7 (1.8–4.0) | 43.0 (39.6–46.3) | 11.9 (9.7–14.5) | 69.1 (65.1–72.8) |
| **Province** |  |  |  |  |
| Province 1 | 4.9 (2.8–8.6) | 31.3 (25.8–37.4) | 6.9 (4.6–10.3) | 54.3 (46.7–61.7) |
| Madhesh province | 3.0 (1.7–5.3) | 50.2 (43.3–57.2) | 5.7 (3.8–8.5) | 73.4 (64.6–80.7) |
| Bagmati Province | 1.9 (0.9–3.7) | 44.0 (39.0–49.2) | 9.4 (5.0–16.8) | 38.4 (32.7–44.4) |
| Gandaki Province | 0.9 (0.3–3.0) | 39.9 (33.1–47.1) | 7.7 (4.7–12.3) | 40.6 (33.6–47.9) |
| Lumbini Province | 2.7 (1.4–5.2) | 46.6 (40.9–52.4) | 16.8 (12.7–21.9) | 61.8 (54.0–68.9) |
| Karnali Province | 1.2 (0.3–4.0) | 46.3 (38.2–54.6) | 11.6 (7.8–16.9) | 80.0 (72.4–85.9) |
| Sudhurpashchim Province | 6.1 (4.0–9.2) | 45.7 (39.9–51.6) | 11.6 (8.6–15.5) | 67.5 (60.3–73.9) |

**Table S2:** Socioeconomic and education-based absolute inequality in the prevalence of suspected developmental delays among Nepalese children aged 3-4 years (N=2,870)

|  | **Socioeconomic inequality** | |  | **Maternal education-based inequality** | |
| --- | --- | --- | --- | --- | --- |
|  | Slope Index of Inequality  (95% CI) | p-value |  | Slope Index of Inequality  (95% CI) | p-value |
| **National level** | -0.32 (-0.37, -0.26) | **<0.001** |  | -0.36 (-0.41, -0.30) | **<0.001** |
| **Place of residence** |  |  |  |  |  |
| Urban area | -0.33 (-0.41, -0.26) | **<0.001** |  | -0.36 (-0.43, -0.28) | **<0.001** |
| Rural area | -0.26 (-0.35, -0.17) | **<0.001** |  | -0.31 (-0.40, -0.22) | **<0.001** |
| **Province** |  |  |  |  |  |
| Province 1 | -0.20 (-0.34, -0.06) | **0.005** |  | -0.33 (-0.46, -0.19) | **<0.001** |
| Madhesh province | -0.27 (-0.42, -0.13) | **<0.001** |  | -0.28 (-0.43, -0.13) | **<0.001** |
| Bagmati Province | -0.37 (-0.49, -0.25) | **<0.001** |  | -0.33 (-0.46, -0.20) | **<0.001** |
| Gandaki Province | -0.12 (-0.28, 0.05) | 0.172 |  | -0.19 (-0.36, -0.03) | **0.024** |
| Lumbini Province | -0.47 (-0.60, -0.34) | **<0.001** |  | -0.44 (-0.59, -0.30) | **<0.001** |
| Karnali Province | -0.37 (-0.65, -0.09) | **0.009** |  | -0.22 (-0.41, -0.03) | **0.022** |
| Sudhurpashchim Province | -0.31 (-0.48, -0.13) | **0.001** |  | -0.13 (-0.31, 0.05) | 0.147 |

**Table S3:** Socioeconomic inequality in the prevalence of suspected delays in physical, social-emotional, learning/cognition, and literacy-numeracy domains among Nepalese children aged 3-4 years (N=2,870)

|  | **Absolute inequality** | |  | **Relative inequality** | | | |
| --- | --- | --- | --- | --- | --- | --- | --- |
|  | Slope Index of Inequality (95% CI) | p-value |  | Relative Index of Inequality (95% CI) | p-value | Concentration Index (95% CI) | p-value |
| Delay in physical domain | | | | | | | |
| **National level** | 0.004 (-0.02, 0.02) | 0.706 |  | 1.14 (0.38, 1.89) | **0.003** | 0.03 (-0.07, 0.14) | 0.571 |
| **Place of residence** |  |  |  |  |  |  |  |
| Urban area | -0.03 (-0.06, -0.002) | **0.034** |  | 0.42 (0.10, 0.75) | **0.010** | -0.12 (-0.24, 0.002) | **0.047** |
| Rural area | 0.02 (-0.02, 0.05) | 0.310 |  | 2.08 (-0.81, 4.97) | 0.159 | 0.12 (-0.08, 0.33) | 0.233 |
| **Province** |  |  |  |  |  |  |  |
| Province 1 | 0.07(0.002, 0.14) | **0.043** |  | 5.20 (-2.30, 12.69) | 0.174 | 0.27 (0.07, 0.48) | **0.008** |
| Madhesh province | -0.045 (-0.11, 0.02) | 0.157 |  | 0.23 (-0.21, 0.66) | 0.306 | -0.19 (-0.42, 0.05) | 0.128 |
| Bagmati Province | -0.02 (-0.06, 0.02) | 0.276 |  | 0.37 (-0.26, 1.00) | 0.254 | -0.16 (-0.41, 0.10) | 0.223 |
| Gandaki Province | -0.02 (-0.06, 0.02) | 0.394 |  | 0.18 (-0.43, 0.79) | 0.568 | -0.30 (-0.73, 0.12) | 0.158 |
| Lumbini Province | 0.01 (-0.05, 0.07) | 0.780 |  | 1.39 (-0.83, 4.61) | 0.397 | -0.31 (-0.73, 0.12) | 0.158 |
| Karnali Province* | N/A |  |  | N/A |  | N/A |  |
| Sudhurpashchim Province | 0.70 (-0.02, 0.16) | 0.114 |  | 3.13 (-1.11, 7.37) | 0.148 | 0.20 (-0.003, 0.40) | 0.054 |
| Delay in social-emotional domain | | | | | | | |
| **National level** | -0.06 (-0.13, 0.002) | 0.059 |  | 0.87 (0.74, 0.99) | **<0.001** | -0.02 (-0.04, 0.005) | 0.115 |
| **Place of residence** |  |  |  |  |  |  |  |
| Urban area | -0.01 (-0.17, 0.004) | **0.039** |  | 0.81 (0.65, 0.97) | **<0.001** | -0.03 (-0.06, 0.001) | **0.046** |
| Rural area | -0.07 (-0.17, 0.02) | 0.137 |  | 0.84 (0.65, 1.03) | **<0.001** | -0.02 (-0.06, 0.01) | 0.221 |
| **Province** |  |  |  |  |  |  |  |
| Province 1 | -0.13 (-0.29, 0.02) | 0.092 |  | 0.65 (0.33, 0.98) | **<0.001** | -0.06 (-0.14, 0.02) | 0.174 |
| Madhesh province | -0.09 (-0.25, 0.07) | 0.249 |  | 0.82 (0.54, 1.01) | **<0.001** | -0.05 (-0.11, 0.001) | **0.048** |
| Bagmati Province | -0.11 (-0.25, 0.04) | 0.165 |  | 0.79 (0.52, 1.05) | **<0.001** | -0.05 (-0.10, 0.003) | 0.061 |
| Gandaki Province | -0.10 (-0.29, 0.09) | 0.288 |  | 0.77 (0.39, 1.14) | **<0.001** | -0.04 (-0.12, 0,04) | 0.379 |
| Lumbini Province | -0.02 (-0.19, 0.15) | 0.822 |  | 0.96 (0.61, 1.31) | **<0.001** | 0.002 (-0.06, 0.06) | 0.944 |
| Karnali Province | -0.01 (-0.30, 0.28) | 0.948 |  | 0.98 (0.32, 1.64) | **0.004** | -0.06 (-0.13, 0.01) | 0.092 |
| Sudhurpashchim Province | 0.03 (-0.15, 0.21) | 0.753 |  | 1.07 (0.63, 1.51) | **<0.001** | -0.04 (-0.10, 0.03) | 0.278 |
| Delay in learning/cognition domain | | | | | | | |
| **National level** | **-0.08 (-0.12, -0.04)** | **<0.001** |  | 0.43 (0.25, 0.61) | **<0.001** | -0.011 (-0.18, 0.05) | **0.001** |
| **Place of residence** |  |  |  |  |  |  |  |
| Urban area | -0.02 (-0.06, 0.03) | 0.486 |  | 0.816 (0.35, 1.28) | **0.001** | -0.004 (-0.10, 0.09) | 0.928 |
| Rural area | -0.13 (-0.20, -0.07) | **<0.001** |  | 0.30 (0.13, 0.48) | **0.001** | -0.173 (-0.26, 0.08) | **<0.001** |
| **Province** |  |  |  |  |  |  |  |
| Province 1 | -0.05 (-0.14, 0.04) | 0.317 |  | 0.54 (-0.10, 1.18) | 0.099 | -0.14 (-0.34, 0.06) | 0.179 |
| Madhesh province | -0.02 (-0.10, 0.05) | 0.514 |  | 0.66 (-0.15, 1.48) | 0.109 | -0.0003 (-0.19, 0.19) | 0.998 |
| Bagmati Province | 0.002 (-0.08, 0.08) | 0.954 |  | 1.04 (-0.24, 2.31) | 0.112 | 0.06 (-0.14, 0.27) | 0.546 |
| Gandaki Province | -0.44 (-0.15, 0.07) | 0.433 |  | 0.57 (-0.23, 1.36) | 0.165 | -0.04 (-0.12, 0.04) | 0.379 |
| Lumbini Province | -0.21 (-0.33, -0.08) | **0.001** |  | 0.28 (0.07, 0.49) | **0.011** | -0.21 (-0.33, -0.09) | **<0.001** |
| Karnali Province | -0.07 (-0.29, 0.14) | 0.515 |  | 0.520 (-0.49, 1.53) | 0.312 | -0.14 (-0.32, 0.03) | 0.110 |
| Sudhurpashchim Province | -0.08 (-0.22, 0.04) | 0.180 |  | 0.44 (-0.08, 0.97) | 0.095 | -0.11 (-0.28, 0.06) | 0.204 |
| Delay in literacy-numeracy domain | | | | | | | |
| **National level** | -0.32 (-0.38, -0.26) | **<0.001** |  | 0.37 (0.30, 0.44) | **<0.001** | -0.16 (-0.17, -0.14) | **<0.001** |
| **Place of residence** |  |  |  |  |  |  |  |
| Urban area | -0.60 (-0.67, -0.54) | **<0.001** |  | 0.27 (0.21, 0.32) | **<0.001** | -0.19 (-0.22, -0.17) | **<0.001** |
| Rural area | -0.33 (-0.41, -0.24) | **<0.001** |  | 0.60 (0.51, 0.69) | **<0.001** | -0.08 (-0.10, -0.06) | **<0.001** |
| **Province** |  |  |  |  |  |  |  |
| Province 1 | -0.49 (-0.63, -0.36) | **<0.001** |  | 0.35 (0.23, 0.48) | **<0.001** | -0.16 (-0.21, -0.11) | **<0.001** |
| Madhesh province | -0.35 (-0.48, -0.21) | **<0.001** |  | 0.61 (0.48, 0.74) | **<0.001** | -0.08 (-0.11, -0.05) | **<0.001** |
| Bagmati Province | -0.58 (-0.69, -0.47) | **<0.001** |  | 0.19 (0.11, 0.27) | **<0.001** | -0.23 (-0.29, -0.17) | **<0.001** |
| Gandaki Province | -0.17 (-0.36, -0.02) | 0.086 |  | 0.67 (0.35, 0.98) | **<0.001** | -0.10 (-0.18, -0.02) | **0.011** |
| Lumbini Province | -0.61 (-0.72, -0.49) | **<0.001** |  | 0.32 (0.22, 0.42) | **<0.001** | -0.16 (-0.20, -0.12) | **<0.001** |
| Karnali Province | -0.55 (-0.71, -0.38) | **<0.001** |  | 0.44 (0.27, 0.60) | **<0.001** | -0.05 (-0.08, -0.02) | **0.004** |
| Sudhurpashchim Province | -0.48 (-0.63, -0.34) | **<0.001** |  | 0.45 (0.31, 0.59) | **<0.001** | -0.12 (-0.16, -0.08) | **<0.001** |

*Sample size was insufficient to perform equity analysis.

**Table S4:** Maternal education-based inequality in the prevalence of suspected delays in physical, social-emotional, learning/cognition, and literacy-numeracy domains among Nepalese children aged 3-4 years (N=2,870)

|  | **Absolute inequality** | |  | **Relative inequality** | | | |
| --- | --- | --- | --- | --- | --- | --- | --- |
|  | **Slope Index of Inequality (95% CI)** | **p-value** |  | **Relative Index of Inequality (95% CI)** | **p-value** | **Concentration Index (95% CI)** | **p-value** |
| Delay in physical domain | | | | | | | |
| **National level** | -0.03 (-0.05, -0.01) | **0.011** |  | 0.035 (0.08, 0.63) | **0.011** | -0.14 (-0.26, -0.02) | **0.02** |
| **Place of residence** |  |  |  |  |  |  |  |
| Urban area | -0.04 (-0.07, -0.01) | **0.016** |  | 0.31 (0.03, 0.58) | **0.032** | -0.16 (-0.3, -0.02) | **0.025** |
| Rural area | -0.03 (-0.06, 0.01) | 0.121 |  | 0.30 (-0.13, 0.74) | 0.174 | -0.17 (-0.38, 0.04) | 0.112 |
| **Province** |  |  |  |  |  |  |  |
| Province 1 | -0.01 (-0.07, 0.06) | 0.889 |  | 0.89 (-0.56, 2.34) | 0.228 | 0.07 (-0.19, 0.33) | 0.593 |
| Madhesh province | -0.10 (-0.21, 0.003) | 0.058 |  | 0.04 (-0.07, 0.15) | 0.461 | -0.30 (-0.52, -0.07) | 0.009 |
| Bagmati Province | -0.06 (-0.13, 0.07) | 0.078 |  | 0.07 (-0.11, 0.25) | 0.441 | -0.37 (-0.65, -0.08) | 0.012 |
| Gandaki Province | 0.01 (-0.03, 0.04) | 0.694 |  | 1.93 (-4.22, 8.09) | 0.538 | -0.02 (-0.49, 0.46) | 0.943 |
| Lumbini Province | -0.002 (-0.05, 0.04) | 0.928 |  | 0.91 (-0.81, 2.64) | 0.299 | -0.01 (-0.34, 0.32) | 0.945 |
| Karnali Province | -0.02 (-0.09, 0.04) | 0.428 |  | 0.15 (-0.56, 0.85) | 0.685 | -0.03 (-0.49, 0.43) | 0.895 |
| Sudhurpashchim Province | -0.01 (-0.09, 0.07) | 0.837 |  | 0.87 (-0.28, 2.02) | 0.139 | 0.05 (-0.17, 0.26) | 0.67 |
| Delay in social-emotional domain | | | | | | | |
| **National level** | -0.08 (-0.15, -0.02) | **0.011** |  | 0.82 (0.69, 0.94) | **<0.001** | -0.02 (-0.05, 0.00) | 0.056 |
| **Place of residence** |  |  |  |  |  |  |  |
| Urban area | -0.08 (-0.17, 0.004) | 0.062 |  | 0.82 (0.655, 0.99) | **<0.001** | -0.03 (-0.06, 0.01) | 0.114 |
| Rural area | -0.09 (-0.19, 0.01) | 0.066 |  | 0.80 (0.61, 0.99) | **<0.001** | -0.02 (-0.06, 0.01) | 0.205 |
| **Province** |  |  |  |  |  |  |  |
| Province 1 | -0.13 (-0.28, 0.03) | 0.108 |  | 0.67 (0.33, 1.00) | **<0.001** | -0.06 (-0.14, 0.02) | 0.174 |
| Madhesh province | -0.08 (-0.25, 0.079) | 0.308 |  | 0.83 (0.54, 1.12) | **<0.001** | -0.05 (-0.11, 0.00) | 0.054 |
| Bagmati Province* | N/A |  |  | N/A |  | -0.02 (-0.08, 0.03) | 0.416 |
| Gandaki Province | -0.20 (-0.39, -0.01) | 0.044 |  | 0.60 (0.29, 0.90) | **<0.001** | -0.07 (-0.15, 0.01) | 0.084 |
| Lumbini Province | -0.14 (-0.31, 0.02) | 0.090 |  | 0.72 (0.45, 1.00) | **<0.001** | -0.03 (-0.09, 0.03) | 0.308 |
| Karnali Province | -0.003 (-0.20, 0.20) | 0.975 |  | 0.99 (0.54, 1.44) | **<0.001** | -0.03 (-0.10, 0.05) | 0.478 |
| Sudhurpashchim Province | -0.01 (-0.09, 0.07) | 0.837 |  | 0.87 (-0.28, 2.02) | 0.139 | 0.01 (-0.05, 0.08) | 0.716 |
| Delay in learning/cognition domain | | | | | | | |
| **National level** | -0.06 (-0.10, -0.02) | **0.004** |  | 0.53 (0.30, 0.76) | **<0.001** | -0.06 (-0.13, 0.01) | 0.081 |
| **Place of residence** |  |  |  |  |  |  |  |
| Urban area | -0.05 (-0.10, -0.0002) | **0.049** |  | 0.54 (0.21, 0.87) | **<0.001** | -0.06 (-0.15, 0.04) | 0.257 |
| Rural area | -0.06 (-0.12, 0.01) | 0.111 |  | 0.61 (0.24, 0.98) | **0.001** | -0.03 (-0.13, 0.06) | 0.454 |
| **Province** |  |  |  |  |  |  |  |
| Province 1 | -0.04 (-0.14, 0.05) | 0.342 |  | 0.55 (-0.12, 1.22) | 0.106 | -0.12 (-0.32, 0.08) | 0.239 |
| Madhesh province | -0.017 (-0.09, 0.06) | 0.662 |  | 0.76 (-0.15, 1.68) | 0.100 | 0.04 (-0.12, 0.21) | 0.61 |
| Bagmati Province | -0.05 (-0.13, 0.03) | 0.239 |  | 0.472 (-0.12, 1.05) | 0.112 | -0.05 (-0.24, 0.14) | 0.61 |
| Gandaki Province | -0.06 (-0.19, 0.07) | 0.355 |  | 0.46 (-0.29, 1.21) | 0.228 | -0.07 (-0.15, 0.01) | 0.084 |
| Lumbini Province | -0.09 (-0.22, 0.05) | 0.207 |  | 0.59 (0.11, 1.07) | **0.017** | -0.13 (-0.26, -0.01) | **0.041** |
| Karnali Province | -0.10 (-0.24, 0.037) | 0.150 |  | 0.39 (-0.10, 0.89) | 0.121 | -0.17 (-0.36, 0.01) | 0.065 |
| Sudhurpashchim Province | -0.05 (-0.17, 0.07) | 0.417 |  | 0.63 (-0.07, 1.33) | 0.076 | -0.07 (-0.25, 0.10) | 0.417 |
| Delay in literacy-numeracy domain | | | | | | | |
| **National level** | -0.54 (-0.59, -0.49) | **<0.001** |  | 0.36 (0.31, 0.40) | **<0.001** | -0.14 (-0.15, -0.12) | **<0.001** |
| **Place of residence** |  |  |  |  |  |  |  |
| Urban area | -0.59 (-0.66--0.53) | **<0.001** |  | 0.28 (0.22, 0.33) | **<0.001** | -0.17 (-0.19, -0.14) | **<0.001** |
| Rural area | -0.40 (-0.48--0.32) | **<0.001** |  | 0.53 (0.45, 0.61) | **<0.001** | -0.09 (-0.11, -0.06) | **<0.001** |
| **Province** |  |  |  |  |  |  |  |
| Province 1 | -0.60 (-0.72--0.48) | **<0.001** |  | 0.26 (0.16, 0.36) | **<0.001** | -0.21 (-0.25, -0.16) | **<0.001** |
| Madhesh province | -0.36 (-0.49--0.23) | **<0.001** |  | 0.59 (0.46, 0.72) | **<0.001** | -0.08 (-0.11, -0.05) | **<0.001** |
| Bagmati Province | -0.51 (-0.64--0.39) | **<0.001** |  | 0.24 (0.14, 0.35) | **<0.001** | -0.22 (-0.28, -0.17) | **<0.001** |
| Gandaki Province | -0.22 (-0.42--0.01) | **0.035** |  | 0.59 (0.30, 0.89) | **<0.001** | -0.13 (-0.21, -0.06) | **0.001** |
| Lumbini Province | -0.55 (-0.67--0.42) | **<0.001** |  | 0.37 (0.25, 0.48) | **<0.001** | -0.15 (-0.19, -0.11) | **<0.001** |
| Karnali Province | -0.31 (-0.46--0.15) | **<0.001** |  | 0.67 (0.51, 0.82) | **<0.001** | -0.06 (-0.09, -0.03) | **0.001** |
| Sudhurpashchim Province | -0.33 (-0.49--0.17) | **<0.001** |  | 0.60 (0.43, 0.77) | **<0.001** | -0.10 (-0.14, -0.05) | **<0.001** |

*Sample size was insufficient to perform equity analysis.

**Table S5**: Variations in the prevalence of suspected developmental delays among Nepalese children before and after adjusting for risk factors (N=2,870)

| **Provinces** | **Prevalence (95% confidence interval)** | |
| --- | --- | --- |
|  | **Crude** | **Adjusted for risk factors^1^** |
| Province 1 | 23.1 (17.7–29.4) | 20.4 (16.6–24.2) |
| Madhesh province | 44.2 (38.2–50.3) | 42.3 (38.1–46.5) |
| Bagmati Province | 26.1 (21.6–31.2) | 32.7 (27.9–37.5) |
| Gandaki Province | 20.5 (15.6–26.5) | 21.0 (14.9–27.1) |
| Lumbini Province | 40.0 (33.5–46.8) | 37.5 (33.1–41.9) |
| Karnali Province | 45.0 (37.4–52.8) | 30.1 (23.0–37.2) |
| Sudhurpashchim Province | 40.1 (34.3–46.1) | 31.8 (26.1–37.5) |

Note: ^1^Adjusted for child’s age, sex, nutritional status (stunting), mother’s education, and household socio-economic status

**References**

1. Loizillon A, Petrowski N, Britto P, Cappa C: Development of the early childhood development index in MICS surveys. MICS Methodological Papers, No. 6. In*.* New York: Data and Analytics Section, Division of Data, Research and Policy, UNICEF; 2017.

2. Central Bureau of Statistics: Nepal multiple indicator cluster survey 2019, Survey Findings Report. In*.* Kathmandu, Nepal: Central Bureau of Statistics and UNICEF Nepal; 2020.

3. World Health Organization: Handbook on health inequality monitoring: with a special focus on low-and middle-income countries: World Health Organization; 2013.

4. Rutstein SO, Johnson K: The DHS wealth index. DHS comparative reports no. 6. In*.* Calverton: ORC Macro; 2004.
